# Effect of Sucrose on Amino Acid Absorption of Whey: A Randomized Crossover Trial

**DOI:** 10.3390/metabo12040282

**Published:** 2022-03-23

**Authors:** Mai Wajiki, Takayuki Yamamoto, Hiroko Maruki-Uchida, Hirotaka Nagashima, Tetsu Shimizu, Minoru Morita

**Affiliations:** 1 Health Science Research Center, Morinaga & Co., Ltd., 2-1-1 Shimosueyoshi, Tsurumi-ku, Yokohama 230-8504, Japan; m-wajiki-ai@morinaga.co.jp (M.W.); t-yamamoto-jc@morinaga.co.jp (T.Y.); m-morita-je@morinaga.co.jp (M.M.); 2 Tokyo Center Clinic, 1-1-8 Yaesu, Chuou-ku, Tokyo 192-0397, Japan; nagashima_hirotaka@tc-clinic.jp (H.N.); t-shimizu@3h-ms.co.jp (T.S.)

**Keywords:** sucrose, whey protein, insulin, amino-acids

## Abstract

Protein intake has been reported to secrete insulin and lower glucose levels, but the effect of carbohydrate and protein co-ingestion on amino acid absorption has not been well documented. A randomized, placebo-controlled, single-blinded, crossover trial was conducted to evaluate the effect of sucrose on blood amino acid levels. Eleven volunteers (both sexes aged 20–60 years with body mass index 21.4 ± 2.4 kg/m^2^) randomly received one of four test solutions: water (P-group), 10 g sucrose (S-group), 10 g whey protein (W-group), or 10 g whey protein + 10 g sucrose (W-S-group), and blood amino acid concentration, glucose levels, and insulin levels were monitored over 180 min. Following the wash-out period, randomized treatment and blood parameter monitoring were repeated. Consequently, amino acid concentration was significantly lower in the S-group than in the P-group, showing that single ingestion of sucrose decreased blood amino acid levels in a fasted state. However, there was no significant difference between blood amino acid levels of the W- and W-S-groups, suggesting that co-ingestion of sucrose does not affect blood amino acid concentration. Insulin levels were significantly higher in the W-S than in the S-group, and glucose levels were significantly lower in the W-S- than in the S-group, suggesting positive impact on glycotoxicity by reducing blood glucose levels. Therefore, whey protein co-ingestion with sucrose suppresses glucose levels and increases insulin levels as opposed to the sucrose ingestion, but does not affect amino acid absorption of whey protein, indicating that this co-ingestion may not be a problem for protein supplementation.

## 1. Introduction

Protein comprises one of the five major nutrient groups, composed of smaller units known as amino acids, which are indispensable for animal and human health. Dietary sources of protein include fish, meat, soybeans, eggs, and dairy products. However, in Japan, protein intake has declined in recent years and is often insufficient [1]. In particular, it is difficult to obtain sufficient dietary protein from the diet during physical training.

Muscle protein synthesis (MPS) rates increase as blood amino acid concentration rises [2]. Conversely, insufficient nutrient consumption leads to muscle protein breakdown (MPB), even in continuous training [3]. Whey protein, the isolated protein from the liquid remaining after fat and casein removal from milk, is widely ingested as a protein supplement following exercise [4]. It is rich in branched-chain amino acids, and its consumption after exercise promotes muscle hypertrophy and strength [5,6]. Whey protein is also reported to improve post-training muscle recovery [7], and it is often taken with carbohydrates because of its taste and recovery ability [8].

Protein intake is also recommended for elderly people because its combination with muscle mass has a dose–response relationship [9], and muscle strength is associated with healthy life [10]. Japan’s Dietary Intake Standards have been revised, and the lower limit of protein intake for elderly people has been raised. Therefore, many confections and other food items with added protein are newly sold in the market. A number of people who consume protein powder or protein confections casually is increasing. However, whether carbohydrate and protein co-ingestion affects amino acid absorption has not been fully investigated for the general population that consumes protein products.

The majority of studies investigating the simultaneous ingestion of carbohydrates and proteins include entire meals [11], and few have explored the relationship between ingestion of isolated whey protein and simple carbohydrates [12]. It has been reported that protein ingestion alters insulin secretion via incretin signaling and lowers blood glucose levels. However, the influence of carbohydrate ingestion on amino acid absorption of protein has not been examined. In general, when there are enough carbohydrates, proteins are used as a material for protein synthesis; thus, protein absorption is not affected by carbohydrates. However, there are no reports of studies in simple situations. Therefore, we analyzed the movement of blood amino acids by comparing it with carbohydrate intake alone. We thought that we would be able to provide certain information to people who consumes protein powder or protein confections. Protein powder and confections usually contain 5–20 g of protein and approximately the same amount of sucrose. Sucrose is the most commonly used carbohydrate, and we thought it was important to explore its impact. Considering the commercial products, the sensitivity of the analysis, and the linearity between amino acid concentrations and protein intake [5], we determined the amount of protein and sucrose as 10 g.

The present study investigated the effect of sucrose co-ingestion on amino acid absorption of whey protein during the fasted state in a wide age range. In addition, the effect of sucrose ingestion on blood amino acid concentration is examined. Glucose levels and insulin levels are also measured.

## 2. Results

### 2.1. Plasma Total Amino Acid (TAA) Concentrations

Twelve participants were enrolled, one excluded herself due to concerns regarding COVID-19 exposure, and 11 healthy adult volunteers (six males and five females) remained. The age range was 20–60 years (average, 34.5 ± 3.0 years for males and 39.8 ± 4.3 years for females). One piece of female data was missing, but mean body height was 169.8 ± 6.1 cm for male and 163.1 ± 5.5 cm for female, mean body weight was 63.3 ± 7.3 kg for males and 54.5 ± 3.1 kg for females, and mean body mass index was 22.0 ± 3.0 kg/m^2^ for males and 20.5 ± 0.3 kg/m^2^ for females.

**Figure 1:**
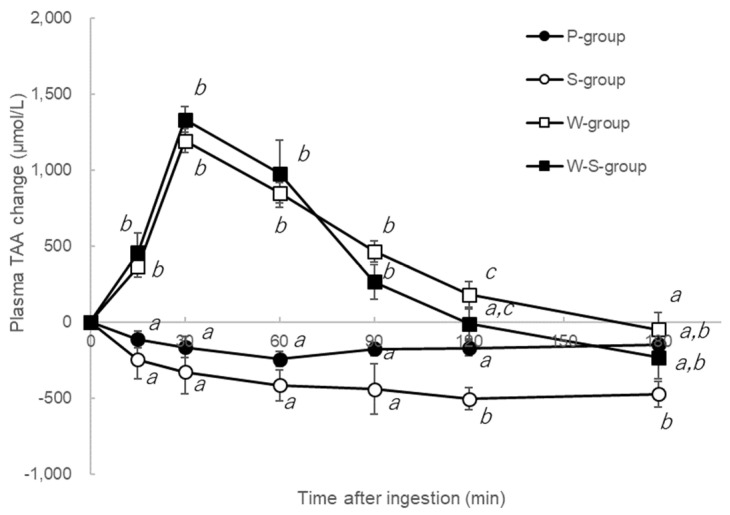
Total amino acid concentrations of P-, S-, W-, and W-S-groups. Each group was measured plasma total amino acid concentrations after ingested test solutions (P: water, S: sucrose, W: whey, and W-S: whey plus sucrose). All values are presented as mean ± SEM. *n* = 11 per group. Means without a common letter at same time point are statistically different (*p* < 0.05, two-way repeated measures ANOVA followed by Tukey’s multiple comparisons).

All plasma TAA concentrations for the P-, S-, W-, and W-S-groups from each baseline are shown in Figure 1. The graph shows the increase in amino acid. At 15–90 min post-ingestion, TAA levels of the W- and W-S-groups were elevated relative to those of the P- and S-groups. Similar trends were noted for plasma essential amino acid (EAA) and branched-chain amino acid (BCAA) levels. At 120 min post-ingestion, TAA levels of the S-group were lower relative to those of the P-group. However, amino acid concentration levels did not differ significantly between the W-S- and W-groups at any point. Furthermore, neither the time taken to achieve the highest TAA concentration (T_max_) nor the area under the curve (AUC) from 0–180 min differed significantly between the W-S- and W-groups (Table 1).

### 2.2. Blood Glucose Levels

**Figure 2:**
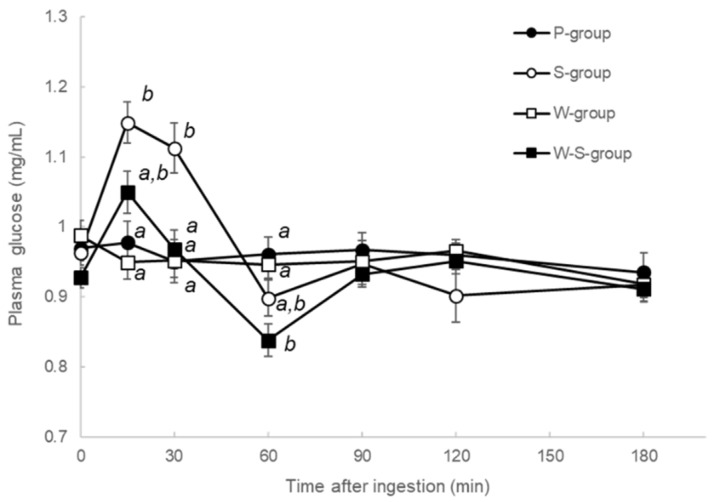
Blood glucose levels of P-, S-, W-, and W-S-groups. Each group was measured plasma glucose levels after ingested test solutions (P: water, S: sucrose, W: whey, and W-S: whey plus sucrose). All values are presented as mean ± SEM. *n* = 11 per group. Means without a common letter at same time point are statistically different (*p* < 0.05, two-way repeated measures ANOVA followed by Tukey’s multiple comparisons).

All blood glucose levels for the P-, S-, W-, and W-S-groups are shown in Figure 2. Between 15 and 30 min post-ingestion, blood glucose levels of the S-group were elevated relative to those of the P- and W-groups. Especially at 30 min after ingestion, blood glucose levels of the S-group were elevated relative to those of the W-S group. As expected, blood glucose levels did not differ significantly between the W- and P-groups.

### 2.3. Plasma Insulin Levels

**Figure 3:**
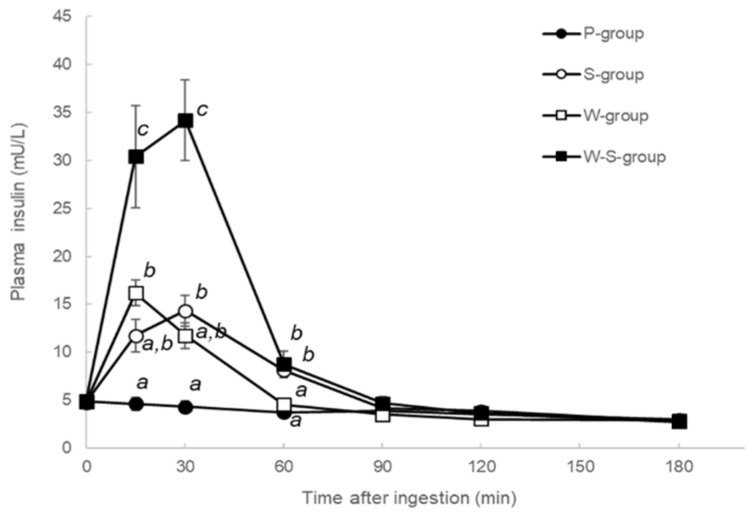
Plasma insulin levels of the P-, S-, W-, and W-S-groups. Each group was measured plasma glucose levels after ingested test solutions (P: water, S: sucrose, W: whey, and W-S: whey plus sucrose). All values are presented as mean ± SEM. *n* = 11 per group. Means without a common letter at same time point are statistically different (*p* < 0.05, two-way repeated measures ANOVA followed by Tukey’s multiple comparisons.

All plasma insulin levels for the P-, S-, W-, and W-S-groups are shown in Figure 3. Between 15 and 30 min post-ingestion, plasma insulin levels of the W-group were elevated relative to those of the other groups, and there was no significant difference between the S- and W-groups. At 60 min post-ingestion, plasma insulin levels of the S- and W-groups were elevated relative to those of the P- and W-groups. There was no significant difference after 90 min.

## 3. Discussion

The present study investigated the effect of sucrose on blood amino acid concentrations. Sucrose co-ingestion with protein in the fasted state did not significantly impact amino acid concentration, T_max_, or AUC. In contrast, sucrose ingestion only significantly decreased amino acid concentration relative to water consumption. There was no difference in blood amino acid levels between the W- and W-S-groups, probably because the increase in amino acids due to whey ingestion was large. As for glucose and insulin, sucrose co-ingestion with protein increased blood insulin levels compared to only sucrose or protein ingestion and decreased blood glucose levels compared to only sucrose or protein ingestion due to insulin secretion. In recent years, protein deficiency has become a concern, and the average human consumes more protein in the form of confections and other foods. The fact that carbohydrates had no effect on blood amino acid levels after protein intake in this study of a wide age range encourages people to consume protein-rich confections and foods.

This study could not show the detailed mechanisms of how glucose affects blood amino acid levels. However, the hormones responsible for regulating nutrient usage may contribute to the mechanisms. During sucrose and protein ingestion, hormones such as insulin and glucagon are released [13]. These hormones impact amino acid metabolism. Insulin favors protein synthesis by enhancing amino acid availability [14]. Participants in the present study fasted prior to nutrient ingestion. During fasting, muscle tissue releases glucogenic amino acids into the blood for conversion to glucose. In the S-group, sucrose-induced insulin secretion may suppress protein degradation, thereby accounting for the observed decrease in blood amino acid concentration in this group. While glucagon promotes skeletal muscle wasting to supply amino acids as gluconeogenic precursors, glucagon secretion also promotes the use of circulating amino acids for gluconeogenesis [15,16,17]. Glucagon may be secreted by sugar ingestion compared to water, but the present study did not monitor glucagon levels.

Compared with previous reports on the simultaneous ingestion of protein and carbohydrates [12,18], it is agreed that blood glucose level is suppressed compared to carbohydrate ingestion alone, and insulin secretion is enhanced compared to carbohydrate ingestion alone. For example, glucose and gelatin (protein) ingestion is reported to show less glucose response than glucose ingestion due to the slowing down of gastric emptying and stimulating incretin hormones and non-glucose-dependent insulin release [12]. It is also reported that protein inclusion in breakfast lowers post-prandial glucose levels [19]. However, insulin level was greater in the present study, and the difference may be caused by carbohydrate sucrose and protein sources or the subject’s attributes. It is possible that taking protein and sucrose at the same time have a synergistic effect on blood insulin levels, but this is not clear from the results of this study. Nowadays, lifestyle-related diseases such as diabetes due to excessive sugar intake are rising. Eating protein confections instead of carbohydrates is not only a good way to make up for the lack of protein but also a good way to avoid rising blood glucose levels. A persistent hyperglycemic state has various deleterious effects including the generation of reactive oxygen species that promote arteriosclerosis and is thus believed to lead to diabetic complications, including myocardial infarction [20,21]. However, insulin secretion has a positive and negative effect on body, so the total long-term effects must be considered separately.

Sucrose and protein co-ingestion also stimulates the release of incretins, such as glucose-dependent insulinotropic polypeptide (GIP) and glucagon-like peptide-1 (GLP-1) [22], which are insulinotropic and known to be involved in the whey-induced amelioration of postprandial glycemia [23]. Therefore, incretins may play an important role in results observed during the present study and should be measured during similar future studies. The present study demonstrated higher plasma insulin levels after co-consumption of whey protein and sucrose relative to the ingestion of sucrose in isolation; incretins may be involved in the mechanism underlying this observation.

There are limitations in this study. First, the study was performed with a small sample size (only 6 men and 5 women) with large age interval (20–60 years, i.e., young and middle-aged subjects mixed). Therefore, age- and gender-differences cannot be analyzed. Second, the habitual physical activity as an important environmental and metabolic factor was not measured in this study. The background of the subjects has not been fully investigated, so it is not possible to show whether there is a relationship between this information and the data.

In conclusion, the present study demonstrates that sucrose consumption in isolation decreases blood amino acid concentration and reports that co-consumption of whey protein with sucrose negates this effect. It also confirms that whey protein and sucrose co-consumption decrease the sucrose-induced increase in blood glucose level while increasing the plasma insulin level. Therefore, our findings indicate that protein co-ingestion with carbohydrates is an effective way to supply protein.

## 4. Materials and Methods

### 4.1. Study Design and Participants

Randomized, Latin square design, single-blinded, placebo-controlled, crossover trial was conducted between January and March 2020. The primary outcome was the change in blood amino acid concentration, and the secondary outcomes were the changes in blood glucose and insulin levels. The study was conducted according to the guidelines of the Declaration of Helsinki and approved by the Japan Conference of Clinical Research (17 January 2020, no. 2020-001). Informed consent was obtained from all the participants involved in the study. The trial was registered in the University Hospital Medical Information Network Clinical Trial Registry of Japan (registration no. UMIN000039301) in January 2020. Dates defining the periods of recruitment and follow-up are 17 January and 31 May in 2020.

**Figure 4:**
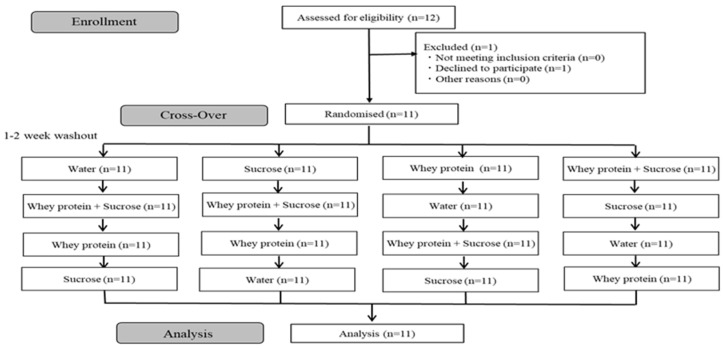
Study flow chart.

Twelve healthy volunteers (seven males and five females; age range was 20–60 years) with no diseases were recruited from the Tokyo Center Clinic by 3H Medi Solution Inc. (Tokyo, Japan). The cohort’s sample size was determined based on a previous report [24]. Figure 4 shows a flow chart of the study. After allocation, none of the participants withdrew or were excluded for the study.

Exclusion criteria are as follows:

(1) Individuals for whom sucrose ingestion and/or whey protein solutions may adversely affect their health.
(2) Individuals who had been hospitalized or were on medications.
(3) Individuals with a history of serious hepatopathy, renal damage, cardiac disease, pulmonary disease, or blood disease.
(4) Individuals who contracted or had a history of serious gastrointestinal disease.
(5) Individuals with an addiction to alcohol or who were mentally unfit to provide informed consent.
(6) Individuals who had used a therapeutic pharmaceutical drug within the preceding month.
(7) Individuals exhibiting symptoms of probable seasonal allergy during the recruitment period. Specifically, this included allergies to *Betulaceae* (alder, oba alnus firma, shirakaba), *Taxodiaceae* (*Cryptomeria* spp., hinoki cypress), *Asteraceae* (ragweed, *Artemisia vulgaris* var. indica), and *Gramineae* (*Dactylis glomerata*, *Phleum pratense*).
(8) Individuals with known severe food and drug allergies, for whom the possibility of a severe allergic reaction to sucrose and/or whey protein ingestion could not be excluded.
(9) Individuals unable to consume milk or dairy products.
(10) Individuals with severe anemia.
(11) Individuals with high fasting blood glucose (>126 mg/dL).
(12) Individuals who are smokers.
(13) Females who are pregnant or lactating.
(14) Individuals expecting a significant lifestyle change during the study period. (Long-term travel, late-night work, etc.)
(15) Individuals who habitually ingested healthy foods and/or supplements within the preceding three months or expecting to do so during the study period.
(16) Individuals who were hospitalized and had received medical treatment within the preceding six months.
(17) Individuals who had participated in other clinical studies within the preceding month or expected to do so during the study period.
(18) Individuals who are not healthy. (Including BMI > = 30 kg/m^2^ considering criteria of Japan Society for the Study of Obesity).
(19) Individuals judged to be inappropriate for study inclusion by the principal investigator.

### 4.2. Experimental Protocols: Nutrient Supplementation and Blood Analysis

Participants fasted (except for water consumption) from 21:00 on the preceding day. At 09:00 on day one of the trial, blood samples were collected at baseline at Tokyo Center Clinic. Participants were then randomized to receive one of four 200 mL test solutions: water (P-group), 10 g sucrose (S-group), 10 g whey protein (W-group), or 10 g whey protein + 10 g sucrose (W-S-group). Additionally, a separate group was set to receive 20 g of whey protein to check the saturation levels; it was later excluded from the study. The assigning list was kept in a sealed envelope by the allocator until the trial was completed, and any person concerned with the study, excluding the allocator, remained blinded; however, the participants could estimate the group by the taste of the administered solution. Therefore, we treated this study as single-blind. Following a one-week wash-out period, participants were again randomized to receive one of the four types of supplement solution, and blood collection was repeated. Thereafter, blood samples were recollected at 15, 30, 60, 90, 120, and 180 min. In more detail, blood samples were collected into sodium heparin plasma vacuettes (TERUMO Corporation, Tokyo, Japan), followed by inversion mixing and centrifugation (1200× *g*, 10 min, 4 °C). To determine the amino acid concentration, plasma was mixed with 6% sulfosalicylic acid solution at a 1:1 ratio and centrifuged (20,400× *g*, 5 min, 15 °C). The supernatant was subjected to derivatization using the AccQ-Tag reagent (Waters Corporation, Milford, UT, USA), followed by measurement of amino acid concentrations using the ACQUITY UPLC H-Class PLUS system (Waters) [25], both according to the manufacturer’s instructions. Glucose levels were measured using the Glucose CII-Test (Wako Pure Chemical Industries, Ltd., Osaka, Japan), according to the manufacturer’s instructions. Insulin levels were measured using an insulin-specific ELISA (Mercodia Inc., Winston-Salem, NC, USA), according to the manufacturer’s instructions.

### 4.3. Preparation of Test Solutions

Test solutions were prepared by Morinaga & Co., Ltd. (Tokyo, Japan). Sucrose (10 g) and/or whey protein (10 g, corresponding to 12.6 g of whey powder) were thoroughly dissolved in 200 mL of mineral water by shaking, and an additional 10 mL of mineral water was used to rinse the residual solution from the shaker. All solutions were ingested within 2 min of preparation. The product information of whey protein powder is shown in Table 2.

### 4.4. Statistical Analysis

All statistical analyses were performed using SPSS version 26 (IBM, Armonk, NY, USA). A two-way repeated-measures ANOVA was used to compare amino acid concentration changes from baseline, glucose level, and insulin level dynamics between the four patient groups (P-group, S-group, W-group, and W-S-group). When significant interactions were observed, Tukey’s post hoc test for multiple comparisons was performed. Differences were considered statistically significant at *p* < 0.05. All data are expressed as mean ± standard error of mean (SEM).

## Figures and Tables

**Table 1:** T_max_ and AUC of the whey and whey and sucrose groups.

	W-Group	W-S-Group	*p*-Value
T_max_ (min)	46.36 ± 7.42	43.64 ± 4.72	0.68
AUC (µmol/L)	78,619.74 ± 13,126.47	66,724.72 ± 18,564.73	0.66

**Table 2:** Total and essential amino acid content of whey powder (a) and nutritional information of the test solutions (b).

(**a**)				
**Amino Acid**			**g/100 g**	
Arginine			2.00	
Lysine			6.13	
Histidine			1.42	
Phenylalanine			2.53	
Tyrosine			2.51	
Leucine			8.77	
Isoleucine			5.28	
Methionine			1.74	
Valine			4.75	
Alanine			4.47	
Glycine			1.41	
Proline			4.47	
Glutamic acid			14.50	
Serine			4.26	
Aspartic acid			8.71	
Cysteine			2.05	
Threonine			5.84	
Tryptophan			1.49	
(**b**)				
**Per Test Solutions**	**P-Group**	**W-Group**	**S-Group**	**W-S-Group**
Energy (kcal)	0.00	46.80	40.00	86.80
Protein (g)	0.00	10.00	0.00	10.00
Fat (g)	0.00	0.75	0.00	0.75
Carbohydrate (g)	0.00	0.00	10.00	10.00

## Data Availability

The data presented in this study are available on request from the corresponding author. Because of the participant consent obtained as part of the recruitment process, it is not possible to make these data publicly available.
